# 0900. Effects of oxygen status on the innate immune response in humans *in vivo*

**DOI:** 10.1186/2197-425X-2-S1-O24

**Published:** 2014-09-26

**Authors:** D Kiers, A John, E Janssen, GJ Scheffer, H van der Hoeven, P Pickkers, M Kox

**Affiliations:** Radboud University Medical Center, Department of Intensive Care Medicine, Nijmegen, Netherlands; Radboud Institute for Infectious Diseases, Nijmegen, Netherlands; Radboud University Medical Center, Department of Anesthesiology, Nijmegen, Netherlands

## Introduction

*In vitro* and animal studies have shown that hypoxia and hyperoxia influence the innate immune response. Therefore, hypoxia and hyperoxia could be cheap, non-pharmacological, non-invasive treatment modalities to modulate inflammatory conditions. Hypoxia has shown to exert pro-inflammatory effects, supposedly mediated by the transcription factor hypoxia inducible factor 1α (HIF1α), whereas hyperoxia is related to immune suppression. However, apart from direct effects of oxygen status adjustment on the immune response, other mechanisms such as a hypoxia-induced stress response might play a role in humans *in vivo* as well. Up till now, the interplay between oxygen status adjustment and the innate immune response in humans *in vivo* has not been investigated.

## Objectives

To evaluate the effects of hypoxia and hyperoxia on the systemic innate immune responseduring experimental endotoxemia in healthy volunteers.

## Methods

We performed a parallel randomized controlled study in 30 healthy male volunteers. Using a non-invasive ventilation helmet, subjects were exposed to a total of 3.5 hours of either hypoxia (mixture of nitrogen and room air titrated to an arterial oxygen saturation of 80-85%, n=10), normoxia (room air, n=10), or hyperoxia (100% oxygen, n=10). Actual FiO_2_ in the helmet was measured using a gas analyzer. One hour after the start of oxygen status adjustment, 2 ng/kg purified *E. Coli* endotoxinwas administered intravenously.

## Results

**Figure 1:**
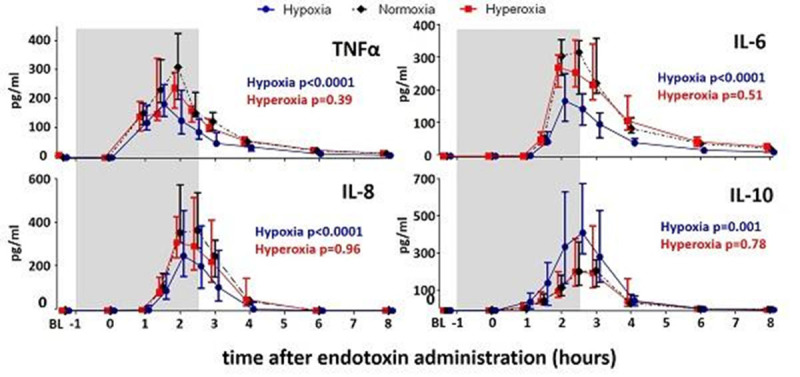
Plasma cytokine levels during hypoxic, normoxic, and hyperoxic endotoxeimia. Data are represented as median with interquartile range of 10 subjects per group. Gray box indicates period of oxygen status adjustment. P-values represent separate comparisons of the hypoxia and hyperoxia group with the normoxic group (two-way ANOVA-repeated measures [interaction term] on the log-transformed data). BL:baseline.

**Figure 2:**
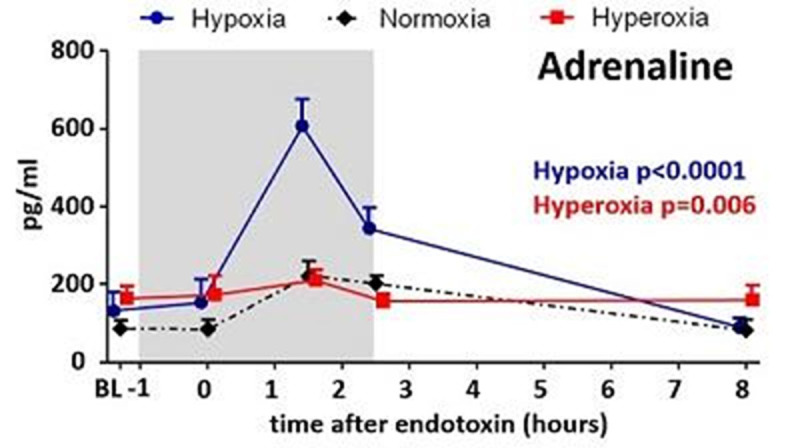
Plasma adrenaline levels during hypoxic, normoxic, and hyperoxic endotoxeimia. Data are represented as means with SEM of10 subjects per group. Gray box indicates period of oxygen status adjustment. P-values represent separate comparisons of the hypoxia and hyperoxia group with the normoxic group (two-way ANOVA-repeated measures [interaction term] on the log-transformed data). BL:baseline.

Both hypoxia and hyperoxia were well-tolerated. An FiO_2_ of 11.5±0.8% was required to induce hypoxia (SaO_2_: 81.9±0.5%, PaO_2_: 5.8 ±0.4 kPa), while hyperoxia (FiO_2_: 97.9±0.2 %) resulted in a mean PaO_2_ of 54.1±4.1kPa. Hypoxia attenuated the endotoxin-induced increase in plasma levels of pro-inflammatory cytokines TNFα, IL-6, and IL-8 , while potentiating the anti-inflammatory IL-10 response (Figure 1). Hyperoxia did not affect cytokine levels. Hypoxia resulted in a profound increase of plasma adrenaline levels, which were not influenced by hyperoxia (Figure 2).Furthermore, inverse correlations between adrenaline and the pro-inflammatory cytokines IL-6 and IL-8 (r=-0.48, p=0.007 and r=-0.47, p=0.004, respectively) and a positive correlation between adrenaline and IL-10 (r=0.52, p=0.004) were found. Finally, intracellular HIF-1α expression was increased in circulating neutrophils 2,5 and 6 hours after endotoxin administration, and 6 hours post-endotoxin in circulating lymphocytes. Hypoxia or hyperoxia did not affect HIF-1α expression.

## Conclusions

In contrast with *in vitro* and animal data, three-and-a-half hours of moderate hypoxia in healthy volunteers attenuates the endotoxin-induced systemic innate immune response, whereas hyperoxia exerts no immunomodulatory effects. The hypoxia-induced immunosuppression is (at least in part) mediated by an increased endogenous adrenaline response. HIF-1α expression increases in circulating leukocytes after endotoxin administration, but is not affected by oxygen status.

